# Comparison of early treatment with ceftolozane/tazobactam versus polymyxin-based therapy of pneumonia due to MDR *Pseudomonas* *aeruginosa* (PUMA)

**DOI:** 10.1128/aac.00569-25

**Published:** 2025-09-18

**Authors:** Thomas P. Lodise, Jae Min, Brian H. Nathanson, Emre Yücel

**Affiliations:** 1 Pharmacy Practice, Albany College of Pharmacy and Health Sciences 1091, Albany, New York, USA; 2 Merck & Co., Inc., Rahway, New Jersey, USA; 3 OptiStatim LLC, Longmeadow, Massachusetts, USA; University of Fribourg, Fribourg, Switzerland

**Keywords:** *Pseudomonas*, polymyxin, ceftolozane/tazobactam, DOOR, outcomes

## Abstract

Ceftolozane/tazobactam and polymyxin-based regimens are frequently used to treat pneumonia caused by multi-drug-resistant *P. aeruginosa* (MDR-PSA). However, comparative data on global clinical outcomes between these therapies are limited. A multi-centered observational study was performed using the PINC AI^TM^ Healthcare Database (2016–2022). The study population included hospitalized patients ≥ 18 years who were diagnosed with pneumonia and had MDR-PSA (defined as non-susceptible to ≥1 agent in ≥3 antimicrobial categories) on a respiratory or blood culture, receipt of ceftolozane/tazobactam or a polymyxin-based regimen within 3 days of index MDR-PSA culture, receipt of ≥2 days of ceftolozane/tazobactam or a polymyxin-based regimen, and without a COVID-19 diagnosis. A Desirability of Outcome Ranking (DOOR) analysis was performed. Components of the DOOR included in-hospital mortality, discharge destination (home vs other), recurrent MDR-PSA pneumonia, receipt of any renal replacement therapy (RRT) post-index culture in RRT-naive patients, and 30-day pneumonia-related readmissions. In total, 186 patients met the study criteria (104 ceftolozane/tazobactam and 82 polymyxin). In the IPW-adjusted DOOR analysis, a ceftolozane/tazobactam-treated patient had a higher probability of a more favorable outcome (DOOR probability: 61.3%; 95% CI: 56.8%, 65.7%). In the DOOR partial credit analyses, a ceftolozane/tazobactam-treated patient had a higher probability of being discharged home alive with no undesirable outcomes than a polymyxin-treated patient (20.2% vs 9.8%, *P* = 0.04). This real-world evidence study of non-COVID-19 patients with MDR-PSA pneumonia suggests that patients treated with ceftolozane/tazobactam have a higher probability of a more favorable outcome compared with patients treated with a polymyxin-based regimen. Further large-scale studies with detailed dosing are needed to validate the findings.

## INTRODUCTION

Pneumonia (PNA) is one of the most common hospital-acquired infections (HAI) in the United States. Comprising hospital-acquired bacterial pneumonia (HABP) and ventilator-associated bacterial pneumonia (VABP), PNA accounts for 22% of all HAIs (1) and is associated with substantial morbidity and mortality (2). Among the wide spectrum of bacterial pathogens causing HABP/VABP, *Pseudomonas aeruginosa* is one of the most frequent causative pathogens (3). In most cases, clinicians empirically treat HABP/VABP due to *P. aeruginosa* with a conventional anti-pseudomonal β-lactam (i.e., meropenem, piperacillin/tazobactam, ceftazidime, or cefepime), but their effectiveness has been limited by the emergence of multi-drug-resistant (MDR) *P. aeruginosa* isolates (4, 5). In a recent US surveillance study of adult intensive care unit (ICU) patients with PNA, the MDR phenotype was observed in 26.0% of *P. aeruginosa* isolates (6). Acknowledging the public health significance of MDR-PSA (MDR-PSA), the Centers for Disease Control and Prevention (CDC) listed it as a serious threat pathogen in its Antimicrobial Resistance Threats Report (4).

Despite its considerable threat to human health (4), there are currently limited clinical data available to define optimal therapies for patients with PNA due to MDR-PSA, and treatment decisions are largely driven by local epidemiology and patient-specific risk factors (3, 7–9). Historically, clinicians have relied on polymyxins, often in combination with a conventional anti-pseudomonal β-lactam with limited or no *in vitro* microbiologic activity, for the treatment of patients with MDR-PSA PNA (10). Newer agents with high *in vitro* microbiologic activity against MDR-PSA and improved safety profiles relative to the polymyxins like ceftolozane/tazobactam are now available and are increasingly used to treat patients for the treatment of adult patients with HABP/VABP due to MDR-PSA (11, 12). Although there has been a shift in prescribing practices from polymyxin-based regimens to agents like ceftolozane/tazobactam for some patients with HABP/VABP due to MDR-PSA across many institutions, limited published clinical comparative studies exist (13–18). More importantly, “within-patient” analyses (19–23) (i.e., analyses that incorporate both the benefits and risks of treatments into a single outcome, providing a global assessment of a patient’s experience) between ceftolozane/tazobactam and polymyxin-based regimens for patients with HABP/VABP due to MDR-PSA are lacking. Given the importance of understanding the association between benefits and harms when determining the utility of a treatment (22), we sought to compare outcomes using the Desirability of Outcome Ranking (DOOR) strategy between non-COVID-19 adult hospitalized patients with MDR-PSA (MDR was defined as non-susceptible to ≥1 agent in ≥3 antimicrobial categories [5]) PNA who received early treatment with either ceftolozane/tazobactam or polymyxin-based regimens across various US hospitals.

## RESULTS

The study disposition of patients is shown in Table S1. In total, 186 patients met the study criteria (104 ceftolozane/tazobactam patients and 82 polymyxin patients) across 78 hospitals. The comparison of baseline characteristics between treatment groups is shown in Table 1. Treatment groups were largely similar at baseline, with a few exceptions. For hospital-level covariates, significant differences in census region and number of hospital beds were observed between treatment groups. For patient-level covariates, the presence of renal failure was more pronounced in the polymyxin group at a *P*-value of <0.05. The median [IQR] treatment duration in the ceftolozane/tazobactam and polymyxin groups was 9 [6, 15] and 7 [5, 13] days, respectively. Among patients in the ceftolozane/tazobactam group (*n* = 104), 29 (27.9%) patients received an aminoglycoside for ≥2 days, and four (3.8%) received a polymyxin for ≥2 days within the first 5 days of ceftolozane/tazobactam. Among patients in the polymyxin group (*n* = 82), 40 (48.8%) received a carbapenem, 13 (15.9%) received cefepime, 9 (11.0%) received piperacillin/tazobactam, and 14 (17.1%) received a newer β-lactam or β-lactam/β-lactam-β-lactamase inhibitor agent (seven received ceftolozane/tazobactam, seven received ceftazidime/avibactam) within the first 5 days of polymyxin treatment. Antibiotic susceptibility results to the treatment of interest received were available in 52 (50%) and 23 (28%) of the ceftolozane/tazobactam- and polymyxin-treated patients, respectively. Among the 52 ceftolozane/tazobactam patients with susceptibility results for ceftolozane/tazobactam, 43 (82.7%) were susceptible. The index MDR-PSA culture was susceptible to either meropenem, cefepime, or piperacillin-tazobactam (TZP) in 30 (57.7%) of the ceftolozane/tazobactam patients with missing ceftolozane/tazobactam susceptibility results. Among the 23 polymyxin patients with susceptibility results for polymyxins, 21 (91.3%) were susceptible. The index MDR-PSA culture was susceptible to either meropenem, cefepime, or TZP in 35 (59.3%) of the polymyxin patients with missing polymyxin susceptibility results.

**TABLE 1:** Comparison of baseline characteristics between treatment groups ^*h*^

	C/T *N* = 104	%	PB *N* = 82	%	*P*-value
Hospital characteristics					
Census region					
Midwest	32	30.8%	21	25.6%	
Northeast	4	3.8%	14	17.1%	0.007
South	65	62.5%	41	50.0%	
West	3	2.9%	6	7.3%	
Number of Beds					
<300	21	20.2%	16	19.5%	
300 to 499	24	23.1%	36	43.9%	0.007
500+	59	56.7%	30	36.6%	
Teaching hospital	69	66.3%	44	53.7%	0.079
Urban location of the hospital	95	91.3%	70	85.4%	0.201
Patient characteristics					
Mean age, years (SD)	60.8 (13.6)		58.5 (17.1)		0.306
Sex: male	73	70.2%	54	65.9%	0.528
Race					
White	70	67.3%	51	62.2%	
Black	25	24.0%	17	20.7%	
Asian	2	1.9%	3	3.7%	0.446
Other	3	2.9%	7	8.5%	
Unknown	4	3.8%	4	4.9%	
Hispanic	2	1.9%	6	7.3%	0.141
Admission source					
Non-healthcare facility (including home)	68	65.4%	43	52.4%	
Clinic	7	6.7%	8	9.8%	
Transfer from SNF, ICF	15	14.4%	15	18.3%	0.494
Transfer from a different hospital	10	9.6%	11	13.4%	
Transfer from another non-acute care facility or other/unknown	4	3.8%	5	6.1%	
Admission source; community vs else					
Community (non-healthcare facility (including home) & clinic	29	27.88%	31	37.80%	0.151
All other sources	75	72.12%	51	62.20%	
Insurance					
Medicare	60	57.7%	57	69.5%	
Medicaid	22	21.2%	15	18.3%	
Managed care	16	15.4%	8	9.8%	0.437
Commercial/worker's comp/self-pay	4	3.8%	2	2.4%	
Other	2	1.9%	0	0.0%	
Charlson comorbidity score					
Mean (SD)	3.4 (2.4)		3.7 (2.3)		0.448
Median (IQR)	3 [2, 5]		4 [2, 5]		0.329
Charlson comorbidities					
Acute myocardial infarction	16	15.4%	18	22.0%	0.250
Congestive heart failure	44	42.3%	25	30.5%	0.098
Peripheral vascular disease	12	11.5%	5	6.1%	0.201
Cerebrovascular disease	17	16.3%	20	24.4%	0.172
Dementia	6	5.8%	6	7.3%	0.670
COPD	41	39.4%	34	41.5%	0.778
Rheumatoid disease	6	5.8%	0	0.0%	0.035
Peptic ulcer disease	3	2.9%	4	4.9%	0.701
Mild liver disease	5	4.8%	4	4.9%	1.000
Diabetes	21	20.2%	20	24.4%	0.493
Diabetes with complications	22	21.2%	19	23.2%	0.742
Hemiplegia or paraplegia	16	15.4%	13	15.9%	0.930
Renal disease	33	31.7%	39	47.6%	0.034
Cancer	8	7.7%	6	7.3%	0.923
Moderate/severe liver disease	3	2.9%	0	0.0%	0.256
Metastatic cancer	2	1.9%	0	0.0%	0.504
AIDS	1	1.0%	2	2.4%	0.584
Hospital LOS prior to index MDR-PSA PNA culture					
Mean (SD)	8.1 (12.4)		11.6 (30.1)		0.283
Median (IQR)	3 [1, 10]		3 [1, 11]		0.462
Hospitalization in the 6 months prior to index MDR-PSA PNA admission	80	76.9%	58	70.7%	0.338
Pre-COVID-19 admission	91	87.5%	75	91.5%	0.386
Residence in ICU on index MDR-PSA PNA culture day	62	59.6%	52	63.4%	0.597
Presence of PSA on a clinical culture prior to MDR-PSA PNA index date during index admission	22	21.2%	20	24.4%	0.600
Presence of a bloodstream infection(s) for non-MDR-PSA pathogens within 30 days of index MDR-PSA PNA culture day during index MDR-PSA PNA admission	10	9.6%	2	2.4%	0.048
Blood as source of index culture	11	10.6%	4	4.9%	0.156
Index DTR-PSA PNA culture	37	35.6%	20	24.4%	0.100
Polymicrobial MDR-PSA PNA culture (excluding Staphylococcus spp./Streptococcus spp.)	22	21.2%	25	30.5%	0.146
Polymicrobial MDR-PSA PNA culture (including Staphylococcus spp./Streptococcus spp.)	31	29.8%	27	32.9%	0.648
Infection type					
nvHABP	35	33.7%	20	24.4%	
vHABP	42	40.4%	38	46.3%	0.388
VABP	27	26.0%	24	29.3%	
Antibiotics received between admission and index culture (not including index culture day)					
Aminoglycoside^*a*^	9	8.7%	10	12.2%	0.428
β-Lactam (older)^*b*^	61	58.7%	54	65.9%	0.316
Fluoroquinolone	11	10.6%	11	13.4%	0.552
Newer β-Lactam or β-Lactam/β-Lactam-β-Lactamase Inhibitor^*c*^	8	7.7%	2	2.4%	0.189
Vancomycin	49	47.1%	42	51.2%	0.578
Daptomycin	4	3.8%	3	3.7%	1.000
Other glycopeptide/glycopeptide-like agents^*d*^	1	1.0%	0	0.0%	1.000
Macrolide^*e*^	9	8.7%	6	7.3%	0.740
Oxazolidones	5	4.8%	10	12.2%	0.066
Sulfa-like agents^*f*^	6	5.8%	6	7.3%	0.670
Tetracycline-like agents^*g*^	8	7.7%	3	3.7%	0.352
Number of antibiotics received between admission and index culture					
0	39	37.5%	25	30.5%	
1	8	7.7%	8	9.8%	
2	27	26.0%	16	19.5%	0.461
3	14	13.5%	17	20.7%	
≥4	16	15.4%	16	19.5%	
Other antibiotics received during the first 5 days of C/T or PB treatment for 2 or more days					
Aminoglycoside*a*	29	27.9%	11	13.4%	0.017
β-Lactam (older)^*b*^	62	59.6%	61	74.4%	0.035
Fluoroquinolone	13	12.5%	8	9.8%	0.557
Newer β-lactam or β-lactam/β-lactam-β-lactamase inhibitor^*c*^	104	100.0%	14	17.1%	N/A*^i^*
Polymyxin	4	3.8%	82	100.0%	N/A
Vancomycin	51	49.0%	33	40.2%	0.231
Daptomycin	9	8.7%	5	6.1%	0.512
Other glycopeptide/glycopeptide-like agents^*d*^	0	0.0%	0	0.0%	1.000
Macrolide*^e^*	3	2.9%	3	2.7%	1.000
Oxazolidones	18	17.3%	18	22.0%	0.426
Sulfa-Like agents*^f^*	7	6.7%	5	6.1%	0.861
Tetracycline-like agents^*g*^	7	6.7%	11	13.4%	0.126

^*a*^ Aminoglycosides included gentamicin, tobramycin, and amikacin.

^*b*^ Older β-lactams included amoxicillin/clavulanate, amoxicillin, ampicillin, ampicillin/sulbactam, aztreonam, bacampicillin, carbenicillin, cefaclor, cefadroxil, cefamandole, cefazolin, cefdinir, cefditoren pivoxil, cefepime, cefixime, cefonicid, cefoperazone, cefotaxime, cefotetan, cefoxitin, cefpodoxime, cefprozil, ceftaroline, ceftibuten, ceftizoxime, ceftriaxone, cephalexin, cephapirin, dicloxacillin, doripenem, ertapenem, imipenem, loracarbef, meropenem, mezlocillin, pencillin, piperacillin, piperacillin/tazobactam, ticarcillin/clavulanate, and ticarcillin.

^*c*^ Newer β-lactam/β-lactam-β-lactamase inhibitor included ceftolozane/tazobactam, ceftazidime/avibactam, imipenem/relebactam, cefiderocol, or meropenem/vaborbactam.

^*d*^ Other glycopeptide/glycopeptide-like agents included dalbavancin, oritavancin, or telavancin.

^*e*^ Macrolides included azithromycin, clarithromycin, erythro/sulfisox, or quinupristin/dalfopristin.

^*f*^ Sulfa-like drugs included sulfamethoxazole (SMX), SMX-trimethoprim (TMP), trimethoprim, or sulfisoxazole.

^*g*^ Tetracycline-like drugs included doxycycline, eravacycline, minocycline, omadacycline, and tigecycline.

^*h*^ MDR-PSA PNA, multi-drug resistant *P. aeruginosa*; PNA, pneumonia; SD, standard deviation; IQR, interquartile range; SNF, skilled nursing facility; ICF, intermediate care facility; COPD, chronic obstructive pulmonary disease; AIDS, acquired immunodeficiency syndrome; LOS, length of stay; ICU, intensive care unit; MV, mechanical ventilation; DTR, difficult-to-treat; nvHABP, non-ventilator associated hospital-acquired bacterial pneumonia; vHABP, ventilator associated hospital-acquired bacterial pneumonia; VABP, ventilator-associated bacterial pneumonia; C/T, ceftolozane/tazobactam; PB, polymyxin.

^*i*^ N/A, Not applicable.

**Fig 1:**
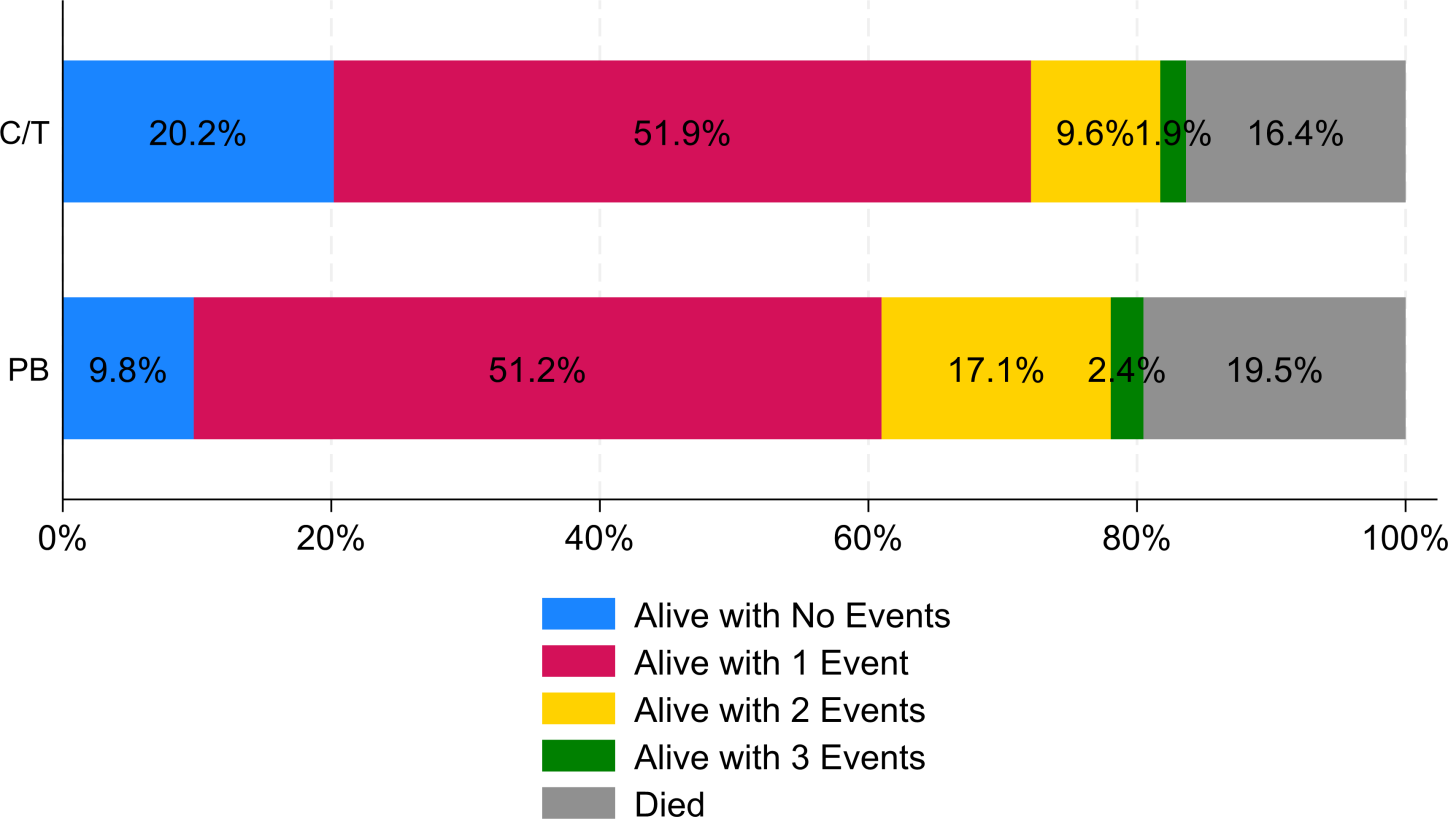
Comparison of desirability of outcome ranking distribution between ceftolozane/tazobactam- and polymyxin-treated patients in the overall study population. Rank one represented the most desirable outcome and included anyone who was discharged alive to home and did not experience any of the undesirable, pre-specified outcomes. Rank six represented the least desirable outcome and included all patients who died during their hospitalization. Ranks 2 through five include patients who were discharged alive but had 1, 2, 3, or 4 events, respectively. The events included in the DOOR analysis were as follows: not discharged home, new receipt of RRT (receipt of RRT after index MDR-PSA culture day in RRT-naïve patients), recurrent MDR-PSA PNA, and 30-day PNA/sepsis-related readmission. Of note, there were no patients discharged alive with four events.

 No significant differences in in-hospital mortality, discharge to home, recurrent MDR-PSA PNA during the index hospitalization, and receipt of any new RRT were observed between treatment groups in the unadjusted and IPW-adjusted analyses (Table 2). However, patients who received ceftolozane/tazobactam treatment were observed to have significantly lower 30-dayPNA/sepsis-related readmissions relative to polymyxin-treated patients (4.9% vs. 15.1%, *P* < 0.05).The distribution of the DOOR rankings between ceftolozane/tazobactam and polymyxin patients in the overall study population is displayed in Fig. 1. The unadjusted DOOR probability was 57.7% (95% CI: 50.1%, 65.3%), indicating that a more desirable DOOR outcome was observed in ceftolozane/tazobactam-treated patients relative to polymyxin-treated patients (Table 3). In the IPW-adjusted DOOR analysis (473 “weighted” observations based on 186 patients), a ceftolozane/tazobactam-treated patient had a 61.3% (95% CI: 56.8%, 65.7%) probability of a more favorable outcome than a polymyxin-treated patient. The probability that a patient in the ceftolozane/tazobactam group would have a more favorable outcome than a patient in the polymyxin group was also observed across the pre-specified subgroups (Table 3). However, the 95% CI associated with each DOOR probability in the subgroup analyses included 50%, indicating no statistically significant differences in DOOR probabilities were observed between treatment groups. Comparison of DOOR partial credit scores between patients who received early ceftolozane/tazobactam or polymyxin-based therapy in the overall study population is shown in Table 4. In patients who value only hospital survival (scenario A), the mean partial credit scores were not significantly different between treatment groups. For patients who prioritize hospital discharge to home and have no undesirable outcomes (scenario B), the mean partial credit score was significantly higher in the ceftolozane/tazobactam group versus the polymyxin group. In patients who prioritize survival but balance it against avoiding some undesirable outcomes (scenario C), the mean partial credit score was higher in the ceftolozane/tazobactam group versus the polymyxin group, but the difference in mean scores was not significant between treatment groups.

**TABLE 2:** Comparison of unadjusted and IPW-adjusted outcomes between ceftolozane/tazobactam and ceftazidime/avibactam in the overall study population^*a*^

Outcomes	C/T *N* = 104	PB *N* = 82	Unadjusted *P*-value	IPW-Adjusted C/T *N* = 104	IPW-Adjusted PB *N* = 82	IPW-Adjusted *P*-value
In-hospital mortality	16.3% (9.8%, 24.9%)	19.5% (11.6%, 29.7%)	0.575	16.8% (9.6%, 24.1%)	20.0% (11.4%, 28.6%)	0.564
Discharged Home vs. other	25.0% (17.0%, 34.4%)	13.4% (6.9%, 22.7%)	0.049	22.5% (14.9%, 30.0%)	14.0% (6.6%, 21.3%)	0.109
Recurrent MDR-PSA pneumonia	5.8% (2.1%, 12.2%)	4.9% (1.3%, 12.0%)	1.000	7.6% (2.0%, 13.2%)	4.8% (0.5%, 9.0%)	0.397
New renal replacement therapy	9.6% (4.7%, 17.0%)	13.4% (6.9%, 22.7%)	0.416	10.6% (4.3%, 16.8%)	12.2% (5.7%, 18.6%)	0.731
30-Day pneumonia/sepsis-related readmission	5.7% (1.9%, 12.9%)	15.2% (7.5%, 26.1%)	0.053	4.9% (0.8%, 9.1%)	15.1% (6.7%, 23.5%)	0.034

^*a*^ C/T, ceftolozane/tazobactam; PB, polymyxin; IPW, inverse probability weighting; MDR-PSA, multi-drug resistant *P. aeruginosa*; IPW, inverse probability weighting.

**TABLE 3:** Comparison of desirability of outcome ranking (DOOR) probabilities between ceftolozane/tazobactam- and polymyxin-treated patients in the overall study population and pre-specified subgroups of interest^*b*^

Group	N^*a*^	DOOR probability (95% CI)
Overall cohort (unadjusted)	186	57.7% (50.1%, 65.3%)
Overall cohort (IPW-adjusted)	473 “weighted” observations based on 186 patients	61.3% (56.8%, 65.7%)
Patients with DTR-PSA PNA	57	59.5% (45.5%, 73.4%)
Patients who resided in an ICU on the index MDR-PSA culture collection day	114	58.1% (48.1%, 68.1%)
Exclusion of patients in the polymyxin group who receive a newer β-lactam or β-lactam/β-lactam-β-lactamase inhibitor agent within the first 5 days of treatment^*a*^	172	55.9% (48.0%, 63.3%)

^*a*^ Patients who received ceftolozane/tazobactam and a polymyxin in the 3 days post index MDR-PSA culture collection day for 2 or more consecutive days were already excluded from the analyses).

^*b*^ IPW, inverse probability weighting; DTR-PSA, difficult-to-treat *P. aeruginosa*; MDR-PSA, multi-drug resistant *P. aeruginosa*; ICU, intensive care unit.

**TABLE 4:** Desirability of outcome ranking (DOOR) partial credit analysis in overall study population^*a*^

Scenarios	Mean C/T score	Mean PB score	Difference in DOOR scores (C/T-PB)	95% CI	Welch *t*-test *P*-value
Scenario A	83.7	80.5	3.2	−8.1, 14.4	0.58
Scenario B	20.2	9.8	10.4	0.3, 20.6	0.04
Scenario C	68.3	62.0	6.3	−3.2, 15.9	0.20

^*a*^ **Scenario A** represents a patient who values only hospital survival (equivalent to a mortality outcome). **Scenario B** represents a patient who prioritizes hospital discharge to home and no undesirable outcomes**. Scenario C** represents a patient who places significant value on survival but balances this with wanting to avoid some undesirable outcomes. For each scenario, the mean of the partial credit scores is calculated for each treatment group, and then the difference between groups is obtained. A difference with a 95% CI that overlaps zero indicates no significant difference between groups.

## DISCUSSION

Limited clinical comparative data are currently available to identify optimal therapies for patients with MDR-PSA PNA (13–17, 24–26). Unlike other therapeutic domains that rely on superiority randomized clinical trials (RCTs) to define best therapies and practices (27, 28), the approval of new agents for the treatment of patients with HABP/VABP has been based predominately on the results of non-inferiority (NI) RCTs (29–32). In addition to the well-described challenges in the design and interpretation of NI-RCTs (28, 33), most HABP/VABP NI-RCTs exclude patients with suspected or documented infections due to pathogens that are resistant to the active comparator to ensure clinical equipoise (29–32, 34). This has important implications for clinical practice, as only scant information on the efficacy of newer agents for the treatment of patients with HABP/VABP caused by highly resistant gram-negative pathogens like MDR-PSA can be ascertained in HABP/VABP NI-RCTs. Several real-world comparative effectiveness studies have been performed between ceftolozane/tazobactam- and polymyxin-based regimens for treatment of adult patients with serious MDR-PSA infections, but the results from these studies reported effectiveness and safety outcomes separately, making it difficult to combine these results into a single global assessment of the patients’ outcomes to inform treatment selection (13–17).

Cognizant of the need for treatment comparisons that reflect the totality of outcomes that matter to both patients and clinicians (19–23), we used the DOOR strategy to compare outcomes between non-COVID-19 adult hospitalized patients with MDR-PSA PNA who received early treatment with either ceftolozane/tazobactam or a polymyxin-based regimen. Our DOOR endpoint included different and important clinical components, including in-hospital mortality, PNA recurrence, receipt of any new RRT, discharge destination (i.e., home versus other), and PNA/sepsis-related readmissions. In the overall analysis, the IPW-adjusted probability that a ceftolozane/tazobactam-treated patient had a more favorable outcome than a polymyxin-treated patient was 61.3% (95% CI: 56.8%, 65.7%). The probability that a patient treated with ceftolozane/tazobactam had a more favorable, although non-significant, outcome compared with a polymyxin-treated patient was also observed among those who were in the ICU on the day of the index MDR-PSA culture collection, had difficult-to-treat (DTR)-*P. aeruginosa* PNA, or only received a conventional anti-pseudomonal β-lactam as part of their polymyxin treatment regimen. From a clinical perspective, the DOOR analysis findings were most effectively captured in the partial credit analyses, which indicated that ceftolozane/tazobactam-treated patients were more likely to be discharged home without undesirable outcomes compared with those treated with polymyxin. In terms of the number needed to treat, the partial credit analysis suggested that for every 9.6 (95% CI: 4.9, 333.3%) MDR-PSA PNA patients treated with a polymyxin-based regimen, one could potentially have experienced the best global outcome (discharge home without undesirable outcomes) if ceftolozane/tazobactam had been administered instead.

The higher probabilities of a more favorable global outcome in ceftolozane/tazobactam-treated patients compared with polymyxin-treated patients were primarily driven by observed differences in discharge destination (home versus other locations), receipt of new renal replacement therapy (RRT), and 30-day PNA/sepsis-related readmissions between the treatment groups (Table 2). No difference in mortality was observed between treatment groups. This finding was not surprising, as death is an insensitive endpoint for differentiating therapies for patients with PNA due to MDR-PSA, given the high underlying risk of death in this population (24, 25, 35–37). More importantly, mortality endpoints do not capture the full spectrum of potential outcomes across the continuum of care that matter to patients and clinicians observed among patients with PNA due to MDR-PSA (24, 25, 35–37). Contrary to other studies (38, 39), no significant difference in receipt of new RRT was observed between treatment groups. The reasons for this are unclear but likely related to the short duration of polymyxin therapy observed in this study. Patients received a median [IQR] of 7 [5, 13] days of polymyxin-based therapy in this study, and data indicate polymyxin-associated AKI increases as a function of treatment duration (40, 41). Unfortunately, we were unable to assess other indicators of acute kidney injury acute kidney injury (e.g., risk, injury, and failure components of the risk of renal dysfunction, injury to kidney, failure or loss of kidney function, and end-stage kidney disease [RIFLE] criteria [42]) that have been shown to be associated with unfavorable short- and long-term deleterious outcomes (43–53) due to the lack of serum creatinine data in most patients in the study cohort. Based on the results of other studies (13, 38, 39, 54–58), it is likely that the incidence of acute kidney injury was higher in the polymyxin group versus the ceftolozane/tazobactam group. As such, the DOOR results should be viewed as a conservative estimate of the differences in global outcomes between treatment groups.

Several limitations should be noted when interpreting the study findings. A high degree of caution should be exercised when interpreting the results, as this was a retrospective observational study. No specific hypotheses were tested, and all analyses were exploratory in nature. Patients were not randomized to treatment, and the potential for systematic biases existed. Study design restrictions and multivariable regression modeling with IPW were used to minimize the influence of the potential confounders (59). However, these methods cannot account for extraneous unmeasured factors that are related to the study outcomes and vary between treatment groups. Thus, this should be viewed as a “hypothesis screening” study, and further large-scale studies are required to fully elucidate the outcomes associated with these treatments for patients with PNA due to MDR-PSA*.*

The limitations of this study also include those inherent to administrative database analyses. Although PHD includes a robust set of hospital- and patient-level data (e.g., diagnostic tests performed, therapeutic services received, medications administered, acuity of hospital care provided [ICU vs. non-ICU], and microbiologic test results) across multiple hospitals (60), clinical laboratory values, physical exam findings, imaging study reports, and physician notes were not available in PHD. We relied on ICD-10-CM codes to define the patients’ baseline disease acuity and infection type, as is common practice in database analyses, but were unable to calculate acute disease severity measures like the Acute Physiology and Chronic Health Evaluation II (APACHE II) and Sequential Organ Failure Assessment (SOFA) scores with the available data in PHD. However, the codes used to identify patients with PNA and their underlying disease severity (i.e., CCI) have been previously validated and shown to have high positive predictive values (61–63). Furthermore, we were able to quantify residence in the ICU on index MDR-PSA PNA culture day, a marker of disease severity, and this was included as a covariate in the IPW-adjusted analyses, and a subgroup analysis was conducted among patients in the ICU. Detailed microbiologic results were available in PHD, but susceptibility data to ceftolozane/tazobactam or a polymyxin were limited, as only a subset of hospitals shared such information with PHD. It is also likely that the susceptibility results for polymyxin were unreliable, given the well-described issues with polymyxin susceptibility testing in clinical laboratories (64). We also had to purposefully limit study outcomes to include only objective measures like MDR-PSA PNA recurrent vs. sepsis/PNA-related readmissions to minimize any subjective biases that may have resulted from assessing clinical response in an electronic healthcare database like PHD. Notably, we relied on receipt of new RRT to identify patients with acute kidney injury, as the serum creatinine data were not available for most patients. We were unable to assess the potential impact of dosing regimens received on the observed treatment outcomes, analyses as only limited data on antibiotic dosing were available. Further large-scale studies with detailed dosing data are required to determine the outcomes associated with these treatments for patients with PNA due to MDR-PSA. Finally, in this study, MDR-PSA was defined as non-susceptibility to ≥1 agent in ≥3 antimicrobial categories, consistent with a previously published definition (5). We acknowledge that different definitions of MDR are used across publications, and readers should consider the specific criteria applied in this study when interpreting the findings.

In conclusion, the findings from this study suggest that non-COVID-19 adult, hospitalized patients with MDR-PSA PNA who received early treatment with ceftolozane/tazobactam had a higher probability of a favorable global outcome than patients who received a polymyxin-based treatment. The results indicate that ~1 out of 10 MDR-PSA PNA patients treated with a polymyxin-based regimen could have potentially been discharged home with no undesirable outcomes (i.e., new RRT, recurrent MDR-PSA PNA and 30-day sepsis/PNA-related 30-day readmission) if ceftolozane/tazobactam versus a polymyxin-based regimen was administered. Further large-scale studies with detailed dosing data are required to determine the outcomes associated with these treatments for patients with PNA due to MDR-PSA.

## MATERIALS AND METHODS

### Study design and population

A retrospective, multi-center observational study of adult (age ≥18 years) hospitalized non-COVID patients in the PINC AI™ Healthcare Data (PHD) (60), between Jan 2016 and Feb 2020 and Jan 2021 and Sep 2022, was performed (hospitalized adult patients in PHD between Mar and Dec 2020 were not included due to poor COVID-19 documentation). PINC AI™ Healthcare Data (PHD), formerly known as the Premier Healthcare Database, is a large, U.S. hospital-based, service-level, all-payer database that contains information on inpatient discharges, primarily from geographically diverse, non-profit, nongovernmental, and community and teaching hospitals and health systems from rural and urban areas. It includes over 121 million inpatient visits with more than 10 million per year since 2012, representing approximately 25% of annual United States inpatient admissions (60). Relevant to this study, the PHD contains information on hospital and visit characteristics, patient demographics and disease states, admission and discharge diagnoses, pharmacy data, microbiologic tests performed, diagnostic and therapeutic services, and patient disposition and discharge health status.

Similar to our recent study (65), patients from PHD during the specified time periods were included in this study if they met the following criteria: (i) evidence of clinical diagnosis for PNA based on International Classification of Diseases, Tenth Revision (ICD-10) diagnostic codes (66), (ii) identification of MDR-PSA, defined as non-susceptible to ≥1 agent in ≥3 antimicrobial categories (5) on a clinical blood or respiratory culture that is consistent with PNA, (iii) receipt of any IV antibiotic(s) 2 days prior to index MDR-PSA culture collection day (−2 days) to ≤3 days after index MDR-PSA collection day (+3 days), (iv) receipt of ceftolozane/tazobactam or a polymyxin within 3 days post-index MDR-PSA culture collection day, and (v) treatment with ceftolozane/tazobactam or a polymyxin for >2 days. Patients were excluded from the study if they had any of the following: (i) diagnosis of cystic fibrosis or moderate to severe bronchiectasis, (ii) hospital length of stay (LOS) < 2 days post index MDR-PSA culture collection day, (iii) transferred from another acute care facility with MDR-PSA on a clinical culture, (iv) missing in-hospital mortality or hospital cost data, (v) simultaneous receipt of ceftolozane/tazobactam and a polymyxin in the 3 days post index MDR-PSA culture collection day for 2 or more consecutive days, (vi) receipt of ceftolozane/tazobactam or a polymyxin more than 2 days before the index culture day for 2 or more consecutive days, and (vii) documented positive SARS-CoV-2 test and/or COVID-19 discharge diagnosis, and those with an inpatient hospitalization with discharge date between 2021 and 2022. Only the first encounter was considered among patients with ≥1 MDR-PSA PNA episodes that met the study criteria during the study period. This study utilized an already existing Health Insurance Portability and Accountability Act (HIPAA)-compliant, fully de-identified data and was exempt from Institutional Review Board (IRB) review (60).

### Baseline data covariates

Hospital-level variables included US census region, hospital bed size, teaching status (teaching vs non-teaching), and population served (urban vs rural). Patient-level variables included demographics, medical history, hospitalization course, microbiology, infection type, and medications received. Patient demographics included age, sex, race, admission source, and primary payer. Medical history included any hospitalizations ≤ 6 months of index admission and the Charlson Comorbidity Index (CCI) (overall score and individual conditions) (67). Data collected during hospital course included hospital LOS prior to index MDR-PSA culture collection day and residence in an ICU on index MDR-PSA culture collection day. Microbiologic data included the presence of *P. aeruginosa* on a clinical culture prior to MDR-PSA culture collection day during index admission; the presence of a bloodstream infection(s) for a non-MDR-PSA pathogen within 30 days of index MDR-PSA culture collection day during the index admission, culture, and antibiotic susceptibility results for index MDR-PSA culture; the presence of DTR (68) or carbapenem resistance on index MDR-PSA culture; the presence of a concurrent MDR-PSA bloodstream infection ±3 days of index MDR-PSA culture; and the presence of other non-MDR-PSA pathogens on index MDR-PSA (i.e., polymicrobial). Pneumonia infection types included non-ventilated HABP (nvHABP), ventilated HABP (vHABP), and VABP (69). Antibiotic(s) received between admission and index MDR-PSA culture collection day, duration of ceftolozane/tazobactam or polymyxin treatment, and other antibiotics received from index ceftolozane/tazobactam or polymyxin treatment day through discharge were documented.

### Outcomes

The primary outcome was a 6-level ranked-ordinal outcome (i.e., DOOR endpoint) and was comprised of the following individual clinical and safety outcomes: in-hospital mortality, discharge destination (home vs other), recurrent MDR-PSA PNA during the index hospitalization, receipt of any RRT post-index MDR-PSA culture collection day among RRT-naive pts (new RRT), and 30-day sepsis/PNA-related readmissions. Each patient was assigned a mutually exclusive rank of 1 through 6 (Table S2). Rank 1 represented the most desirable outcome and included anyone who was discharged alive to home and did not experience any of the individual clinical and safety outcomes. Rank 6 represented the least desirable outcome and included all patients who died during their hospitalization. Ranks 2 through 5 include patients who were discharged alive but experienced 1, 2, 3, or 4 individual study outcomes (i.e., not discharged home, recurrent MDR-PSA PNA during the index hospitalization, receipt of any new RRT, and 30-day sepsis/PNA-related readmissions), respectively.

### Statistical methods

Bivariate analyses were first performed to compare baseline characteristics between treatment groups and individual clinical and safety outcomes between treatment groups. The Student’s *t*-test and the Kruskal-Wallis test were used to compare mean and median continuous variables, respectively, between the two treatment groups. The χ^2^ test was used to compare categorical dependent variables between treatment groups unless a cell count was <5, wherein the Fisher exact test was used. Inverse probability weighting (IPW) was then used to estimate each outcome difference between ceftolozane/tazobactam and polymyxins using a set of plausible confounders determined a priori (Table S3) (70, 71).

A DOOR analysis (22) was performed to determine the probability of a more desirable rank-ordered ordinal outcome with ceftolozane/tazobactam or polymyxin treatment in the overall population and pre-specified subgroups of interest. The probability of a more desirable outcome with one treatment compared with the other (DOOR probability; Wilcoxon Mann–Whitney *U* statistic adjusted for tie) was calculated with a corresponding 2-sided 95% CI (CI) (72). Inverse probability weighting (IPW) (70, 71) was also used to evaluate the difference in the probability of the DOOR outcome between treatment groups while adjusting for potential baseline confounding variables (Table S3) (73). Additionally, subgroup analyses were performed among (i) patients with DTR-PSA PNA (68), (ii) patients in the ICU on index MDR-PSA culture collection day, and (iii) patients in the polymyxin group who did not receive a newer β-lactam or β-lactam/β-lactam-β-lactamase inhibitor agent within the first 5 days of treatment (N.B., patients who received ceftolozane/tazobactam and a polymyxin in the 3 days post-index MDR-PSA culture collection day for 2 or more consecutive days were already excluded from the analyses). A partial credit analysis using three different patient perspective scenarios (i.e., A, B, or C) was also performed (Table S4). Scenario A represents patients who value only hospital survival (equivalent to the in-hospital mortality outcome). Scenario B represents patients who prioritize hospital discharge to home and no undesirable outcomes. Scenario C represents patients who prioritize survival but balance survivorship against avoiding some undesirable outcomes. For each scenario, the mean of the partial credit scores was calculated for each treatment group, and then, the difference between groups was calculated. A difference with a 95% CI that overlaps zero indicates no significant difference between groups for a given scenario. All analyses were done using Stata/MP 18.0 for Windows (StataCorp LLC, College Station, TX). *P*-values < 0.05 were considered statistically significant.

## Data Availability

The data used in this study were obtained from PINC AI and are subject to licensing restrictions. Due to our contractual agreement with PINC AI, we are unable to share the data set publicly. Researchers interested in accessing PINC AI data must obtain a license directly from PINC AI.

## SUPPLEMENTAL MATERIAL

Supplemental tables aac.00569-25-s0001.docx Tables S1 to S4.

ASM does not own the copyrights to Supplemental Material that may be linked to, or accessed through, an article. The authors have granted ASM a non-exclusive, world-wide license to publish the Supplemental Material files. Please contact the corresponding author directly for reuse.

## References

[1] Magill SS, Edwards JR, Bamberg W, Beldavs ZG, Dumyati G, Kainer MA, Lynfield R, Maloney M, McAllister-Hollod L, Nadle J, Ray SM, Thompson DL, Wilson LE, Fridkin SK, Emerging Infections Program Healthcare-Associated Infections and Antimicrobial Use Prevalence Survey Team. 2014. Multistate point-prevalence survey of health care-associated infections. N Engl J Med 370:1198–1208. doi:10.1056/NEJMoa1306801 24670166 PMC4648343

[2] Zilberberg MD, Nathanson BH, Puzniak LA, Shorr AF. 2022. Descriptive epidemiology and outcomes of nonventilated hospital-acquired, ventilated hospital-acquired, and ventilator-associated bacterial pneumonia in the United States, 2012-2019. Crit Care Med 50:460–468. doi:10.1097/CCM.0000000000005298 34534129 PMC8855942

[3] Weiner LM, Webb AK, Limbago B, Dudeck MA, Patel J, Kallen AJ, Edwards JR, Sievert DM. 2016. Antimicrobial-resistant pathogens associated with healthcare-associated infections: summary of data reported to the national healthcare safety network at the centers for disease control and prevention, 2011–2014. Infect Control Hosp Epidemiol 37:1288–1301. doi:10.1017/ice.2016.174 27573805 PMC6857725

[4] Anonymous. Centers for Disease Control and Prevention (CDC). 2019. Antibiotic resistance threats in the United States. Atlanta, GA: U.S. Department of Health and Human Services, CDC. Available from: www.cdc.gov/DrugResistance/Biggest-Threats.html

[5] Magiorakos AP, Srinivasan A, Carey RB, Carmeli Y, Falagas ME, Giske CG, Harbarth S, Hindler JF, Kahlmeter G, Olsson-Liljequist B, Paterson DL, Rice LB, Stelling J, Struelens MJ, Vatopoulos A, Weber JT, Monnet DL. 2012. Multidrug-resistant, extensively drug-resistant and pandrug-resistant bacteria: an international expert proposal for interim standard definitions for acquired resistance. Clin Microbiol Infect 18:268–281. doi:10.1111/j.1469-0691.2011.03570.x 21793988

[6] Sader HS, Castanheira M, Mendes RE, Flamm RK. 2018. Frequency and antimicrobial susceptibility of Gram-negative bacteria isolated from patients with pneumonia hospitalized in ICUs of US medical centres (2015-17). J Antimicrob Chemother 73:3053–3059. doi:10.1093/jac/dky279 30060117

[7] Kalil AC, Metersky ML, Klompas M, Muscedere J, Sweeney DA, Palmer LB, Napolitano LM, O’Grady NP, Bartlett JG, Carratalà J, El Solh AA, Ewig S, Fey PD, File TM Jr, Restrepo MI, Roberts JA, Waterer GW, Cruse P, Knight SL, Brozek JL. 2016. Management of adults with hospital-acquired and ventilator-associated pneumonia: 2016 clinical practice guidelines by the infectious diseases society of America and the American thoracic society. Clin Infect Dis 63:e61–e111. doi:10.1093/cid/ciw353 27418577 PMC4981759

[8] Mensa J, Barberán J, Soriano A, Llinares P, Marco F, Cantón R, Bou G, González Del Castillo J, Maseda E, Azanza JR, Pasquau J, García-Vidal C, Reguera JM, Sousa D, Gómez J, Montejo M, Borges M, Torres A, Alvarez-Lerma F, Salavert M, Zaragoza R, Oliver A. 2018. Antibiotic selection in the treatment of acute invasive infections by Pseudomonas aeruginosa: guidelines by the spanish society of chemotherapy. Rev Esp Quimioter 31:78–100. 29480677 PMC6159363

[9] Bassetti M, Vena A, Croxatto A, Righi E, Guery B. 2018. How to manage Pseudomonas aeruginosa infections. DIC 7:1–18. doi:10.7573/dic.212527 PMC5978525 29872449

[10] Obritsch MD, Fish DN, MacLaren R, Jung R. 2004. National surveillance of antimicrobial resistance in Pseudomonas aeruginosa isolates obtained from intensive care unit patients from 1993 to 2002. Antimicrob Agents Chemother 48:4606–4610. doi:10.1128/AAC.48.12.4606-4610.2004 15561832 PMC529178

[11] Paul M, Carrara E, Retamar P, Tängdén T, Bitterman R, Bonomo RA, de Waele J, Daikos GL, Akova M, Harbarth S, Pulcini C, Garnacho-Montero J, Seme K, Tumbarello M, Lindemann PC, Gandra S, Yu Y, Bassetti M, Mouton JW, Tacconelli E, Rodríguez-Baño J. 2022. European society of clinical microbiology and infectious diseases (ESCMID) guidelines for the treatment of infections caused by multidrug-resistant Gram-negative bacilli (endorsed by European society of intensive care medicine). Clin Microbiol Infect 28:521–547. doi:10.1016/j.cmi.2021.11.025 34923128

[12] Tamma PD, Aitken SL, Bonomo RA, Mathers AJ, van Duin D, Clancy CJ. 2022. Infectious diseases society of America 2022 guidance on the treatment of extended-spectrum β-lactamase producing Enterobacterales (ESBL-E), carbapenem-resistant Enterobacterales (CRE), and Pseudomonas aeruginosa with difficult-to-treat resistance (DTR-P. aeruginosa). Clin Infect Dis 75:187–212. doi:10.1093/cid/ciac268doi:35439291 PMC9890506

[13] Pogue JM, Kaye KS, Veve MP, Patel TS, Gerlach AT, Davis SL, Puzniak LA, File TM, Olson S, Dhar S, Bonomo RA, Perez F. 2020. Ceftolozane/tazobactam vs polymyxin or aminoglycoside-based regimens for the treatment of drug-resistant Pseudomonas aeruginosa. Clin Infect Dis 71:304–310. doi:10.1093/cid/ciz816 31545346

[14] Chen J, Liang Q, Chen X, Wu J, Wu Y, Teng G, Huang M. 2022. Ceftazidime/avibactam versus polymyxin B in the challenge of carbapenem-resistant Pseudomonas aeruginosa infection. Infect Drug Resist 15:655–667. doi:10.2147/IDR.S350976 35241917 PMC8887910

[15] Vena A, Giacobbe DR, Mussini C, Cattelan A, Bassetti M, Ceftabuse Study G. 2020. Clinical efficacy of ceftolozane-tazobactam versus other active agents for the treatment of bacteremia and nosocomial pneumonia due to drug-resistant Pseudomonas aeruginosa. Clin Infect Dis 71:1799–1801. doi:10.1093/cid/ciaa003 31904819

[16] Puzniak L, Dillon R, Palmer T, Collings H, Enstone A. 2021. Real-world use of ceftolozane/tazobactam: a systematic literature review. Antimicrob Resist Infect Control 10:68. doi:10.1186/s13756-021-00933-8 33832545 PMC8027296

[17] Caffrey AR, Appaneal HJ, Liao JX, Piehl EC, Lopes V, Dillon RJ, Puzniak LA, LaPlante KL. 2022. The comparative effectiveness of ceftolozane/tazobactam versus aminoglycoside- or polymyxin-based regimens in multi-drug-resistant Pseudomonas aeruginosa infections. Antibiotics (Basel) 11:626. doi:10.3390/antibiotics11050626 35625270 PMC9137796

[18] Pinilla-Rello A, Huarte-Lacunza R, Magallón-Martínez A, Cazorla-Poderoso L, Pereira-Blanco O, Pérez-Moreno M, Larrodé-Leciñena I, Martínez-Álvarez RM, López-Calleja AI. 2021. Utilization study in real clinical practice of ceftolozane/tazobactam vs aminoglycosides and/or colistin in the treatment of multirresistant or extremely resistant Pseudomonas aeruginosa. Rev Esp Quimioter 34:441–449. doi:10.37201/req/006.2021 34154319 PMC8638843

[19] Doernberg SB, Tran TTT, Tong SYC, Paul M, Yahav D, Davis JS, Leibovici L, Boucher HW, Corey GR, Cosgrove SE, Chambers HF, Fowler VG, Evans SR, Holland TL, Antibacterial Resistance Leadership Group. 2019. Good studies evaluate the disease while great studies evaluate the patient: development and application of a desirability of outcome ranking endpoint for Staphylococcus aureus bloodstream infection. Clin Infect Dis 68:1691–1698. doi:10.1093/cid/ciy766 30321315 PMC6495020

[20] Evans SR, Follmann D. 2016. Using outcomes to analyze patients rather than patients to analyze outcomes: a step toward pragmatism in benefit:risk evaluation. Stat Biopharm Res 8:386–393. doi:10.1080/19466315.2016.1207561 28435515 PMC5394932

[21] Evans SR, Rubin D, Follmann D, Pennello G, Huskins WC, Powers JH, Schoenfeld D, Chuang-Stein C, Cosgrove SE, Fowler VG Jr, Lautenbach E, Chambers HF. 2015. Desirability of outcome ranking (DOOR) and response adjusted for duration of antibiotic risk (RADAR). Clin Infect Dis 61:800–806. doi:10.1093/cid/civ495 26113652 PMC4542892

[22] Howard-Anderson J, Hamasaki T, Dai W, Collyar D, Rubin D, Nambiar S, Kinamon T, Hill C, Gelone SP, Mariano D, Baba T, Holland TL, Doernberg SB, Chambers HF, Fowler VG Jr, Evans SR, Boucher HW. 2023. Improving traditional registrational trial end points: development and application of a desirability of outcome ranking end point for complicated urinary tract infection clinical trials. Clin Infect Dis 76:e1157–e1165. doi:10.1093/cid/ciac692 36031403 PMC10169394

[23] Howard-Anderson J, Earley M, Hamasaki T, Bower CW, Smith G, van Duin D, Evans SR, Jacob JT. 2021. 1219. Unfavorable clinical outcomes with polymyxins compared to ceftolozane/tazobactam for the treatment of carbapenem-resistant Pseudomonas aeruginosa. Open Forum Infect Dis 8:S699–S700. doi:10.1093/ofid/ofab466.1411

[24] Decruyenaere J, de Deyne C, Poelaert J, Colardyn F. 1995. Morbidity or mortality as endpoint for clinical trials in intensive care. Lancet 345:986–987. doi:10.1016/s0140-6736(95)90733-5 7715314

[25] Vincent JL. 2010. We should abandon randomized controlled trials in the intensive care unit. Crit Care Med 38:S534–S538. doi:10.1097/CCM.0b013e3181f208ac 21164394

[26] Almangour TA, Ghonem L, Alassiri D, Aljurbua A, Al Musawa M, Alharbi A, Almohaizeie A, Almuhisen S, Alghaith J, Damfu N, Aljefri D, Alfahad W, Khormi Y, Alanazi MQ, Alsowaida YS. 2023. Ceftolozane-tazobactam versus ceftazidime-avibactam for the treatment of infections caused by multidrug-resistant pseudomonas aeruginosa: a multicenter cohort study. Antimicrob Agents Chemother 67:e0040523. doi:10.1128/aac.00405-23 37404159 PMC10433809

[27] Freidlin B, Korn EL, George SL, Gray R. 2007. Randomized clinical trial design for assessing noninferiority when superiority is expected. J Clin Oncol 25:5019–5023. doi:10.1200/JCO.2007.11.8711 17971602

[28] Kishore K, Mahajan R. 2020. Understanding superiority, noninferiority, and equivalence for clinical trials. Indian Dermatol Online J 11:890–894. doi:10.4103/idoj.IDOJ_130_20 33344335 PMC7734976

[29] European Medicines Agency. 2018. Guideline on the evaluation of medicinal products indicated for treatment of bacterial infections. European Medicines Agency. Available from: https://www.ema.europa.eu/en/documents/scientific-guideline/draft-guideline-evaluation-medicinal-products-indicated-treatment-bacterial-infections-revision-3_en.pdf. Retrieved 16 Jul 2018.

[30] United States Food and Drug Administration. 2010. Guidance for industry: antibacterial drug products: use of noninferiority trials to support approval. US Food and Drug Administration. Available from: https://www.fda.gov/media/71215/download. Accessed 16 Jul 2010.

[31] United States Food and Drug Administration. 2020. Guidance for industry: hospital-acquired bacterial pneumonia and ventilator-associated bacterial pneumonia: developing drugs for treatment. US Food and Drug Administration. Available from: https://www.fda.gov/media/79516/download. Accessed 28 Jul 2020.

[32] United States Food and Drug Administration. 2016. Guidance for Industry: Hospital-acquired bacterial pneumonia and ventilator-associated bacterial pneumonia: developing drugs for treatment. US Food and Drug Administration. Available from: https://www.fda.gov/media/78504/download. Retrieved 28 Jul 2016.

[33] Mauri L, D’Agostino RB Sr. 2017. Challenges in the design and Interpretation of noninferiority trials. N Engl J Med 377:1357–1367. doi:10.1056/NEJMra1510063 28976859

[34] Spellberg B, Talbot G, Infectious Diseases Society of A, American College of Chest P, American Thoracic S, Society of Critical Care M. 2010. Recommended design features of future clinical trials of antibacterial agents for hospital‐acquired bacterial pneumonia and ventilator‐associated bacterial pneumonia. CLIN INFECT DIS 51:S150–S170. doi:10.1086/653065 20597666 PMC2947853

[35] Kress JP. 2015. Mortality is the only relevant outcome in ARDS: no. Intensive Care Med 41:144–146. doi:10.1007/s00134-014-3563-6 25476982

[36] Ospina-Tascón GA, Büchele GL, Vincent J-L. 2008. Multicenter, randomized, controlled trials evaluating mortality in intensive care: doomed to fail? Crit Care Med 36:1311–1322. doi:10.1097/CCM.0b013e318168ea3e 18379260

[37] Petros AJ, Marshall JC, van Saene HK. 1995. Should morbidity replace mortality as an endpoint for clinical trials in intensive care? Lancet 345:369–371. doi:10.1016/s0140-6736(95)90347-x 7772121

[38] Wagenlehner F, Lucenteforte E, Pea F, Soriano A, Tavoschi L, Steele VR, Henriksen AS, Longshaw C, Manissero D, Pecini R, Pogue JM. 2021. Systematic review on estimated rates of nephrotoxicity and neurotoxicity in patients treated with polymyxins. Clin Microbiol Infect:S1198-743X(20)30764-3. doi:10.1016/j.cmi.2020.12.009 33359542

[39] Sisay M, Hagos B, Edessa D, Tadiwos Y, Mekuria AN. 2021. Polymyxin-induced nephrotoxicity and its predictors: a systematic review and meta-analysis of studies conducted using RIFLE criteria of acute kidney injury. Pharmacol Res 163:105328. doi:10.1016/j.phrs.2020.105328 33276108

[40] Phe K, Lee Y, McDaneld PM, Prasad N, Yin T, Figueroa DA, Musick WL, Cottreau JM, Hu M, Tam VH. 2014. In vitro assessment and multicenter cohort study of comparative nephrotoxicity rates associated with colistimethate versus polymyxin B therapy. Antimicrob Agents Chemother 58:2740–2746. doi:10.1128/AAC.02476-13 24566187 PMC3993221

[41] Sorlí L, Luque S, Grau S, Berenguer N, Segura C, Montero MM, Alvarez-Lerma F, Knobel H, Benito N, Horcajada JP. 2013. Trough colistin plasma level is an independent risk factor for nephrotoxicity: a prospective observational cohort study. BMC Infect Dis 13:380. doi:10.1186/1471-2334-13-380 23957376 PMC3765824

[42] Bellomo R, Ronco C, Kellum JA, Mehta RL, Palevsky P, Acute Dialysis Quality Initiative workgroup. 2004. Acute renal failure - definition, outcome measures, animal models, fluid therapy and information technology needs: the second international consensus conference of the acute dialysis quality initiative (ADQI) group. Crit Care 8:R204–12. doi:10.1186/cc2872 15312219 PMC522841

[43] Chertow GM, Burdick E, Honour M, Bonventre JV, Bates DW. 2005. Acute kidney injury, mortality, length of stay, and costs in hospitalized patients. J Am Soc Nephrol 16:3365–3370. doi:10.1681/ASN.2004090740 16177006

[44] Abdalrahim MS, Khalil AA, Alramly M, Alshlool KN, Abed MA, Moser DK. 2020. Pre-existing chronic kidney disease and acute kidney injury among critically ill patients. Heart Lung 49:626–629. doi:10.1016/j.hrtlng.2020.04.013 32354485

[45] Bagshaw SM, George C, Dinu I, Bellomo R. 2008. A multi-centre evaluation of the RIFLE criteria for early acute kidney injury in critically ill patients. Nephrol Dial Transplant 23:1203–1210. doi:10.1093/ndt/gfm744 17962378

[46] Bouchard J, Acharya A, Cerda J, Maccariello ER, Madarasu RC, Tolwani AJ, Liang X, Fu P, Liu ZH, Mehta RL. 2015. A prospective international multicenter study of AKI in the intensive care unit. Clin J Am Soc Nephrol 10:1324–1331. doi:10.2215/CJN.04360514 26195505 PMC4527019

[47] Hong D, Ren Q, Zhang J, Dong F, Chen S, Dong W, Chen X, Chen L, Yao Y, Lu Z, Zhao G. 2023. A new criteria for acute on preexisting kidney dysfunction in critically ill patients. Ren Fail 45:2173498. doi:10.1080/0886022X.2023.2173498 36728812 PMC9897760

[48] Hoste EAJ, Bagshaw SM, Bellomo R, Cely CM, Colman R, Cruz DN, Edipidis K, Forni LG, Gomersall CD, Govil D, Honoré PM, Joannes-Boyau O, Joannidis M, Korhonen A-M, Lavrentieva A, Mehta RL, Palevsky P, Roessler E, Ronco C, Uchino S, Vazquez JA, Vidal Andrade E, Webb S, Kellum JA. 2015. Epidemiology of acute kidney injury in critically ill patients: the multinational AKI-EPI study. Intensive Care Med 41:1411–1423. doi:10.1007/s00134-015-3934-7 26162677

[49] Hoste EAJ, Kellum JA, Selby NM, Zarbock A, Palevsky PM, Bagshaw SM, Goldstein SL, Cerdá J, Chawla LS. 2018. Global epidemiology and outcomes of acute kidney injury. Nat Rev Nephrol 14:607–625. doi:10.1038/s41581-018-0052-0 30135570

[50] Kellum JA, Prowle JR. 2018. Paradigms of acute kidney injury in the intensive care setting. Nat Rev Nephrol 14:217–230. doi:10.1038/nrneph.2017.184 29355173

[51] Lebiedz P, Knickel L, Engelbertz C, Lüders F, Gebauer K, Berdel WE, Waltenberger J, Reinecke H. 2014. Impact of preexisting chronic kidney disease on acute and long-term outcome of critically ill patients on a medical intensive care unit. J Nephrol 27:73–80. doi:10.1007/s40620-013-0016-1 24519865

[52] Pickkers P, Darmon M, Hoste E, Joannidis M, Legrand M, Ostermann M, Prowle JR, Schneider A, Schetz M. 2021. Acute kidney injury in the critically ill: an updated review on pathophysiology and management. Intensive Care Med 47:835–850. doi:10.1007/s00134-021-06454-7 34213593 PMC8249842

[53] Singbartl K, Kellum JA. 2012. AKI in the ICU: definition, epidemiology, risk stratification, and outcomes. Kidney Int 81:819–825. doi:10.1038/ki.2011.339 21975865

[54] Doremus C, Marcella SW, Cai B, Echols RM. 2022. Utilization of colistin versus β-lactam and β-lactamase inhibitor agents in relation to acute kidney injury in patients with severe gram-negative infections. Infect Dis Ther 11:187–199. doi:10.1007/s40121-021-00556-x 34731456 PMC8564277

[55] McKinnell JA, Dwyer JP, Talbot GH, Connolly LE, Friedland I, Smith A, Jubb AM, Serio AW, Krause KM, Daikos GL, Group CS. 2019. Plazomicin for infections caused by carbapenem-resistant Enterobacteriaceae. N Engl J Med 380:791–793. doi:10.1056/NEJMc1807634 30786196

[56] Motsch J, Murta de Oliveira C, Stus V, Köksal I, Lyulko O, Boucher HW, Kaye KS, File TM Jr, Brown ML, Khan I, Du J, Joeng H-K, Tipping RW, Aggrey A, Young K, Kartsonis NA, Butterton JR, Paschke A. 2020. RESTORE-IMI 1: a multicenter, randomized, double-blind trial comparing efficacy and safety of imipenem/relebactam vs colistin plus imipenem in patients with imipenem-nonsusceptible bacterial infections. Clin Infect Dis 70:1799–1808. doi:10.1093/cid/ciz530 31400759 PMC7156774

[57] Wunderink RG, Giamarellos-Bourboulis EJ, Rahav G, Mathers AJ, Bassetti M, Vazquez J, Cornely OA, Solomkin J, Bhowmick T, Bishara J, Daikos GL, Felton T, Furst MJL, Kwak EJ, Menichetti F, Oren I, Alexander EL, Griffith D, Lomovskaya O, Loutit J, Zhang S, Dudley MN, Kaye KS. 2018. Effect and safety of meropenem–vaborbactam versus best-available therapy in patients with carbapenem-resistant Enterobacteriaceae infections: The TANGO II randomized clinical trial. Infect Dis Ther 7:439–455. doi:10.1007/s40121-018-0214-1 30270406 PMC6249182

[58] van Duin D, Lok JJ, Earley M, Cober E, Richter SS, Perez F, Salata RA, Kalayjian RC, Watkins RR, Doi Y, Kaye KS, Fowler VG Jr, Paterson DL, Bonomo RA, Evans S, Antibacterial Resistance Leadership Group. 2018. Colistin versus ceftazidime-avibactam in the treatment of infections due to carbapenem-resistant Enterobacteriaceae. Clin Infect Dis 66:163–171. doi:10.1093/cid/cix783 29020404 PMC5850032

[59] Curtis LH, Hammill BG, Eisenstein EL, Kramer JM, Anstrom KJ. 2007. Using inverse probability-weighted estimators in comparative effectiveness analyses with observational databases. Med Care 45:S103–7. doi:10.1097/MLR.0b013e31806518ac 17909367

[60] Anonymous. 2021. PINC AI healthcare data white paper: data that informs and performs. PINC AI Applied Sciences, Premier Inc. Available from: https://offers.premierinc.com/rs/381-NBB-525/images/Premier-HealthcareDatabase-Whitepaper-Final.pdf. Retrieved 14 Sep 2021.

[61] Lagu T, Lindenauer P. 2015. Can we make performance measures more resilient to the effects of coding? Crit Care Med 43:1141–1143. doi:10.1097/CCM.0000000000000897 25876116 PMC4400863

[62] Rothberg MB, Pekow PS, Priya A, Lindenauer PK. 2014. Variation in diagnostic coding of patients with pneumonia and its association with hospital risk-standardized mortality rates: a cross-sectional analysis. Ann Intern Med 160:380–388. doi:10.7326/M13-1419 24723078 PMC6946057

[63] Sjoding MW, Iwashyna TJ, Dimick JB, Cooke CR. 2015. Gaming hospital-level pneumonia 30-day mortality and readmission measures by legitimate changes to diagnostic coding. Crit Care Med 43:989–995. doi:10.1097/CCM.0000000000000862 25746747 PMC4617210

[64] Ezadi F, Ardebili A, Mirnejad R. 2019. Antimicrobial susceptibility testing for polymyxins: challenges, issues, and recommendations. J Clin Microbiol 57:e01390-18. doi:10.1128/JCM.01390-18 30541939 PMC6440778

[65] Lodise TP, Obi EN, Watanabe AH, Yucel E, Min J, Nathanson BH. 2024. Comparative evaluation of early treatment with ceftolozane/tazobactam versus ceftazidime/avibactam for non-COVID-19 patients with pneumonia due to multidrug-resistant Pseudomonas aeruginosa. J Antimicrob Chemother 79:2954–2964. doi:10.1093/jac/dkae313 39258877 PMC11531822

[66] Lodise TP, Puzniak LA, Chen LH, Tian Y, Wei R, Im TM, Tartof SY. 2021. Outcomes of adult patients in the intensive care unit with Pseudomonas aeruginosa pneumonia who received an active anti-pseudomonal β-lactam: does “S” equal success in the presence of resistance to other anti-pseudomonal β-lactams? Pharmacotherapy 41:658–667. doi:10.1002/phar.2600 34097763 PMC8457199

[67] Deyo RA, Cherkin DC, Ciol MA. 1992. Adapting a clinical comorbidity index for use with ICD-9-CM administrative databases. J Clin Epidemiol 45:613–619. doi:10.1016/0895-4356(92)90133-8 1607900

[68] Kadri SS, Adjemian J, Lai YL, Spaulding AB, Ricotta E, Prevots DR, Palmore TN, Rhee C, Klompas M, Dekker JP, Powers JH 3rd, Suffredini AF, Hooper DC, Fridkin S, Danner RL, National Institutes of Health Antimicrobial Resistance Outcomes Research Initiative (NIH–ARORI). 2018. Difficult-to-treat resistance in gram-negative bacteremia at 173 US hospitals: retrospective cohort analysis of prevalence, predictors, and outcome of resistance to all first-line agents. Clin Infect Dis 67:1803–1814. doi:10.1093/cid/ciy378 30052813 PMC6260171

[69] Kollef MH, Nováček M, Kivistik Ü, Réa-Neto Á, Shime N, Martin-Loeches I, Timsit J-F, Wunderink RG, Bruno CJ, Huntington JA, Lin G, Yu B, Butterton JR, Rhee EG. 2019. Ceftolozane–tazobactam versus meropenem for treatment of nosocomial pneumonia (ASPECT-NP): a randomised, controlled, double-blind, phase 3, non-inferiority trial. Lancet Infect Dis 19:1299–1311. doi:10.1016/S1473-3099(19)30403-7 31563344

[70] Cattaneo MD. 2010. Efficient semiparametric estimation of multi-valued treatment effects under ignorability. J Econom 155:138–154. doi:10.1016/j.jeconom.2009.09.023

[71] Chesnaye NC, Stel VS, Tripepi G, Dekker FW, Fu EL, Zoccali C, Jager KJ. 2022. An introduction to inverse probability of treatment weighting in observational research. Clin Kidney J 15:14–20. doi:10.1093/ckj/sfab158 35035932 PMC8757413

[72] Halperin M, Hamdy MI, Thall PF. 1989. Distribution-free confidence intervals for a parameter of Wilcoxon-Mann-Whitney type for ordered categories and progressive censoring. Biometrics 45:509–521. 2765635

[73] Harrell FE. 2001. Regression modeling strategies: with applications to linear models, logistic regression, and survival analysis. springer, New York.

