# Supplementary material for: Comparison of early treatment with ceftolozane/tazobactam versus polymyxin-based therapy of pneumonia due to MDR Pseudomonas aeruginosa (PUMA)
Source: Antimicrob Agents Chemother. 2025 Sep 18;69(11):e00569-25. doi: 10.1128/aac.00569-25 (PMC12587572; doi:10.1128/aac.00569-25)
Supplement: Supplemental tables — Tables S1 to S4. [file aac.00569-25-s0001.docx]

**Supplemental Tables**

**Supplemental Table 1.** Study Attrition

| **Inclusion criteria** | **N** |
| --- | --- |
| Documented inpatient hospitalization with discharge date between Jan 2016- Feb 2020 (pre-COVID-19 period) and Jan 2021-Sep 2022 (post COVID-19 period). Note period between Mar-Dec 2020 was not included due to poor COVID-19 documentation & Age ≥ 18 years old and hospitals are submitting laboratory values. | 1,996,780 |
| Evidence of clinical diagnosis of pneumonia (PNA) by ICD-10 codes | 304,720 |
| Patient has *Pseudomonas aeruginosa* based on laboratory results | 33,287 |
| Patient has a valid respiratory or blood culture source | 24,177 |
| Patient has multiple drug resistance in their respiratory or blood culture P. *aeruginosa* | 5,012 |
| Patient had a valid Index Culture specimen day (e.g., culture was drawn as an inpatient) | 4,960 |
| Receipt of any IV antibiotic(s) 2 days prior to index MDR-PSA culture collection day (-2 days) to ≤ 3 days after index MDR-PSA collection day (+3 days). | 4,150 |
| Receipt of C/T or PB within 5 days post-index MDR-PSA PNA culture collection day and treatment with C/Tor PB was continued for >2 days. | 609 |
| **Exclusion Criteria** |  |
| Diagnosis of cystic fibrosis or moderate to severe bronchiectasis per ICD-10 codes | 526 |
| Patients with missing in-hospital mortality or hospital cost data. | 522 |
| Hospital LOS <2 days post index MDR-PSA culture collection day | 522 |
| Patient transferred from another acute care facility with an index MDR-PSA culture within -1 to +3 days of hospital admission | 485 |
| Patients with multiple encounters after meeting all the inclusion/exclusion criteria above are excluded (i.e., the analysis only uses the first encounter among patients with multiple encounters). | 451 |
| Patients with a documented inpatient hospitalization with discharge date between 2021-2022 and a documented positive SARS-CoV-2 test and or COVID-19 discharge diagnosis. COVID-19 patients will be excluded to minimize the potential of selection bias and confounding in outcome analyses. | 432 |
| Patients who (1) received both C/T and PB within 5 days post-index MDR-PSA PNA culture collection day, (2) C/T and PB were continued for >2 days, and (3) C/T and PB were received for same number of days during the 5-day post-index MDR-PSA PNA culture collection day window. | 430 |
| Patients who received either C/T or PB more than 2 days before the index day for 2 or more consecutive days | 409 |
| Patients who first received C/T or PB more than 2 days after the index date | 186 |

**Supplemental Table 2.** Six-Level Rank Ordered Ordinal Outcome for the DOOR Analyses

| **Rank** | **Alive at hospital discharge** | **How many of the following events:**  **1. Not discharged to home**  **2. New receipt of RRT**  **3. Recurrent MDR-PSA PNA**  **4. 30-day PNA-related readmission** |
| --- | --- | --- |
| 1 (most desirable) | Yes, and discharged to home | 0 of 3 |
| 2 | Yes | 1 of 4 |
| 3 | Yes | 2 of 4 |
| 4 | Yes | 3 of 4 |
| 5 | Yes | 4 of 4 |
| 6 (least desirable) | No (death) | Any |

**Abbreviations:** RRT: renal replacement therapy, MDR: multi-drug resistant, PSA: *P. aeruginosa,* PNA: pneumonia

Rank 1 represented the most desirable outcome and included anyone who was discharged alive to home and did not experience any of the undesirable, pre-specified outcomes. Rank 6 represented the least desirable outcome and included all patients who died during their hospitalization. Ranks 2 through 5 include patients who were discharged alive but had 1, 2, 3, or 4 events, respectively. The events included in the DOOR analysis were as follows: not discharged home, new receipt of renal replacement therapy RRT, defined as receipt of renal RRT after index MDR-PSA culture day in RRT-naïve patients), recurrent MDR-PSA PNA, and 30-day PNA/sepsis-related readmission.

**Supplemental Table 3.** Variables included in the Inverse Probability Weighting Analysis

| Age |
| --- |
| Sex |
| Race |
| Admission Source |
| Charlson Comorbidity Index |
| Hospital LOS from Admission to Index MDR-PSA Culture Day |
| Residence in ICU on Index MDR-PSA Culture Day |
| Infection Type (nvHABP, vHABP, VABP) |
| Presence of a Concurrent MDR-PSA Bloodstream Infections ± 3 Days of Index MDR-PSA Culture Day |
| Presence of a Polymicrobial Infection |
| Index MDR-PSA Culture met DTR Criteria |
| Index MDR-PSA was carbapenem resistance |

**Supplemental Table 4.** DOOR Partial Credit Scoring in Scenarios A, B, and C

| Door Rank | Scenario A (full credit) | Scenario B (no credit) | Scenario C (partial credit) |
| --- | --- | --- | --- |
| Alive and no events | 100 | 100 | 100 |
| Alive with 1 event | 100 | 0 | 80 |
| Alive with 2 events | 100 | 0 | 60 |
| Alive with 3 events | 100 | 0 | 40 |
| Alive with 4 events | 100 | 0 | 20 |
| Death | 0 | 0 | 0 |

**Abbreviations:** MDR-PSA: multi-drug resistant *P. aeruginosa*, PNA: pneumonia, RRT: renal replacement therapy.

**Scenario A** represents a patient who values only hospital survival (equivalent to a mortality outcome).

**Scenario B** represents a patient who places more value on minimizing events and would not accept any undesirable outcomes.

**Scenario C** represents a patient who places significant value on survival but balances this with wanting to avoid some undesirable outcomes.
